# Hematopoietic cell transplantation and gene therapy for Diamond-Blackfan anemia: state of the art and science

**DOI:** 10.3389/fonc.2023.1236038

**Published:** 2023-09-11

**Authors:** Senthil Velan Bhoopalan, Shruthi Suryaprakash, Akshay Sharma, Marcin W. Wlodarski

**Affiliations:** ^1^ Department of Hematology, St. Jude Children’s Research Hospital, Memphis, TN, United States; ^2^ Department of Bone Marrow Transplantation and Cellular Therapy, St. Jude Children’s Research Hospital, Memphis, TN, United States; ^3^ Department of Oncology, St. Jude Children’s Research Hospital, Memphis, TN, United States

**Keywords:** Diamond-Blackfan anemia, DBA, anemia, HCT, HSCT, gene therapy, RPS19, ribosomopathy

## Abstract

Diamond-Blackfan anemia (DBA) is one of the most common inherited causes of bone marrow failure in children. DBA typically presents with isolated erythroid hypoplasia and anemia in infants. Congenital anomalies are seen in 50% of the patients. Over time, many patients experience panhematopoietic defects resulting in immunodeficiency and multilineage hematopoietic cytopenias. Additionally, DBA is associated with increased risk of myelodysplastic syndrome, acute myeloid leukemia and solid organ cancers. As a prototypical ribosomopathy, DBA is caused by heterozygous loss-of-function mutations or deletions in over 20 ribosomal protein genes, with *RPS19* being involved in 25% of patients. Corticosteroids are the only effective initial pharmacotherapy offered to transfusion-dependent patients aged 1 year or older. However, despite good initial response, only ~20-30% remain steroid-responsive while the majority of the remaining patients will require life-long red blood cell transfusions. Despite continuous chelation, iron overload and related toxicities pose a significant morbidity problem. Allogeneic hematopoietic cell transplantation (HCT) performed to completely replace the dysfunctional hematopoietic stem and progenitor cells is a curative option associated with potentially uncontrollable risks. Advances in HLA-typing, conditioning regimens, infection management, and graft-versus-host-disease prophylaxis have led to improved transplant outcomes in DBA patients, though survival is suboptimal for adolescents and adults with long transfusion-history and patients lacking well-matched donors. Additionally, many patients lack a suitable donor. To address this gap and to mitigate the risk of graft-versus-host disease, several groups are working towards developing autologous genetic therapies to provide another curative option for DBA patients across the whole age spectrum. In this review, we summarize the results of HCT studies and review advances and potential future directions in hematopoietic stem cell-based therapies for DBA.

## Introduction

Diamond-Blackfan anemia (DBA) is an intriguing and enigmatic disease that has held the attention of clinicians and scientists alike for close to a century. First described as congenital hypoplastic anemia in 1936 (1, 2), it is characterized by macrocytic anemia, reticulocytopenia, erythroblastopenia, and constitutional anomalies including craniofacial, skeletal, and cardiac abnormalities in about 50% of the patients (3). DBA is an ultra-rare disease, with an incidence of 2-8 per million live births per year (3, 4). Approximately 90% of the patients are diagnosed in the first year of life with 35% presenting in the first 4 months of life with progressive anemia (3, 5). The phenotypic presentation is diverse and can widely vary in between family members with the same mutation (5). Despite profound improvements in our understanding of the molecular mechanisms of this disorder, no newer therapies have met clinical approval since original report of corticosteroids as an effective option in 1951 (6).

## Pathophysiology and genetics

DBA is a ribosomopathy caused by loss-of-function mutations or deletions involving ribosomal protein genes (5). Out of the 83 ribosomal protein (RP) genes, 23 RP genes have so far been reported to be associated with DBA, where the clinical features are secondary to impaired ribosomal biogenesis (3). *RPS19* mutations account for 25% of cases. Very rarely, mutations in non-RP genes such as *EPO* and *GATA1* have been reported cause a DBA-like disease characterized by erythroblastopenia, without any impairment ribosomal biogenesis (5). Several studies have partially elucidated the mechanisms of DBA-associated anemia. DBA bone marrow shows increased apoptosis and impaired maturation of erythroid progenitors (7–10). Potential explanations for these erythroid defects include reduced translation of the essential erythroid transcription factor *GATA1* (11), build-up of cytotoxic free heme due to reduced translation of globin proteins (12), and activation of TP53 via several potential mechanisms (9, 13, 14). In addition to a specific erythroid failure seen in younger patients, bone marrow hypocellularity, progressive multilineage cytopenias and immunodeficiency are noted in older patients with DBA, suggesting impairment of hematopoietic stem and progenitor cells (HSPCs) (15–17). Mechanisms of DBA HSPCs dysfunction are not clear, although TP53 activation appears to play a critical role (18). Additionally, systematic review of published case reports suggested an increased risk of cancer in DBA, which was subsequently confirmed by pioneering studies lead by the DBA Registry of North America (DBAR) (19–21). These prospective studies established DBA as a cancer predisposition syndrome with increased risk of MDS/AML and solid tumors such as colon carcinoma and osteosarcoma (20, 22, 23). While the exact mechanisms underlying this risk of malignancy are unknown, acquired somatic mutations leading to clonal hematopoiesis have been reported in DBA (24). For a more thorough review of the DBA pathophysiology, the readers are referred to recent review articles by Da Costa et al. (3, 5, 25 *)*

## Current treatment options

**Figure 1:**
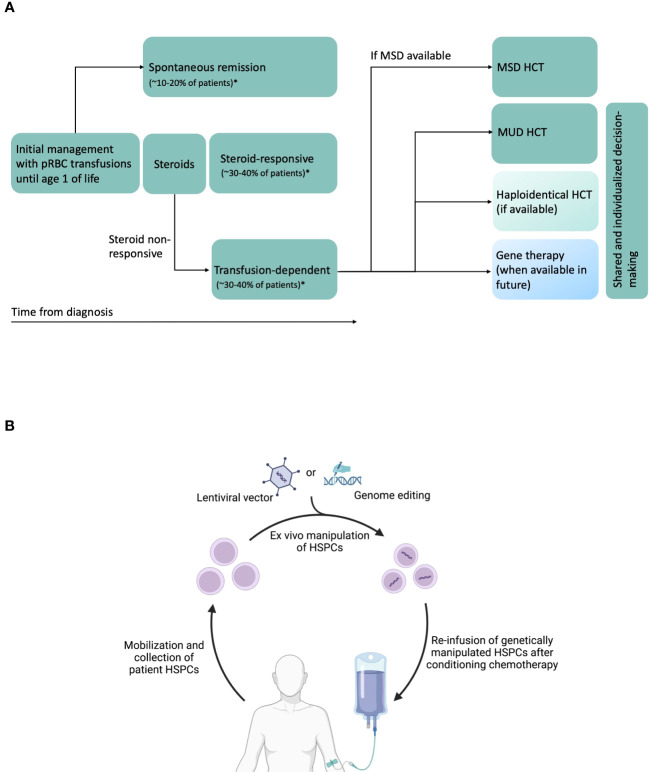
**(A)** Schematic of DBA natural history, and current and potential future therapies. Transfusion-dependent patients are eligible for HCT. Current standards are to perform MSD (preferred) or MUD HCT. Haploidentical HCT can be performed in the absence of MSD or MUDs, preferably on a clinical trial. In future, gene therapy could be another potential therapeutic option. In patients without MSD, treatment decisions will have to be individualized, shared between physicians, patients and patient family members, and consider availability of newer approaches such as haploidentical HCT and gene therapy in the future. *Data from DBA Registry of North America (26) and unpublished data from German DBA registry. **(B)** Schematic of future gene therapy approaches using autologous HSPCs. CD34^+^ HSPCs are mobilized from bone marrow using agents such as G-CSF and plerixafor, collected by apheresis, purified, modified ex vivo with lentiviral vectors or undergo genome editing using novel agents such as CRISPR/Cas9, base editors or prime editors, and infused back into the patient after administration of conditioning chemotherapy. Created with BioRender.com.

The three major therapeutic options for anemia in DBA are packed red blood cell (pRBC) transfusions, corticosteroids (prednisone), and allogeneic hematopoietic cell transplantation (HCT) (**Figure 1A**) (27). Majority of patients are diagnosed during their infancy and initially supported with pRBC transfusions during the first year of life after which corticosteroid treatment is initiated. Oral corticosteroids have been the mainstay of DBA treatment for well over half a century. Oral prednisone is started at a dose of 2 mg/kg/day (or 80 mg/day in adults) for a 2-week period and then tapered over several weeks to months to a maximum maintenance dose of 0.3 mg/kg/day. Although 60-80% of the patients respond to corticosteroids initially, at least half of them will either lose responsiveness or develop steroid intolerance over time (27). Patients who require a maintenance dose > 0.3 mg/kg/day or those that do not respond to prednisone are supported with chronic pRBC transfusions. A second trial of prednisone may be attempted at least 12 months after the first attempt as anecdotally some children may subsequently become responders (27). Prompt evaluation and treatment for iron overloading are critical in transfusion-dependent patients to avoid secondary organ damage and to improve outcomes for a potential HCT.

## Investigational medical therapies

Advances in the understanding of the pathobiology underlying DBA has led to newer medical therapies which are currently being investigated. L-leucine has been shown to improve translational efficiency and activate mTOR pathway resulting in improvement of anemia in animal and cell models of DBA and was recently demonstrated to be safe with modest efficacy in patients with DBA (28–30). Recent studies have suggested that RP deficiency can lead to reduced globin chain production in erythroid cells, resulting in accumulation of free heme that is cytotoxic to erythroid progenitors and precursors (12, 31). Based on this hypothesis, eltrombopag is predicted to improve erythropoiesis in DBA by chelating intracellular iron. Preliminary studies were affected by eltrombopag-induced thrombocytosis and showed low response rate (32). Alternate approaches to inhibit heme accumulation, such as glycine transporter 1 inhibitors (33), are currently being explored (34). Trifluoperazine, which was shown to improve erythroid defect in experimental DBA models by reducing TP53 activity via calmodulin inhibition, is being evaluated for clinical use (14).

## Transplant indications and outcomes

Allogeneic HCT remains the only option for hematopoietic cure in patients with DBA. The first HCT for DBA was performed in 1976, proving that hematological cure is possible (35). Numerous case reports since then confirmed the feasibility of HCT in DBA. The current accepted recommendations for HCT in DBA include transfusion dependence, non-responsiveness to steroids, requiring > 0.3 mg/kg/day of steroids, unacceptable adverse effects from steroid therapy, clinically relevant cytopenias such as neutropenia and transfusion-dependent thrombocytopenia, and evolution to hematologic malignancy (36).

The earliest published data from DBAR showed 87% overall survival (OS) after HCT from HLA-matched related donors (MRD) (37). However, in this early report OS after alternative donor HCTs was only 14%. Multiple retrospective studies, across the globe, have since confirmed the good outcomes after MRD HCT (**Table 1**) (38, 41–44, 46). Outcomes for HLA-matched unrelated donor (MUD) HCTs have shown improvement over the last two decades, mainly due to advances in supportive care, high-resolution HLA typing and better donor-recipient matching. More recent data from the Franco-German DBA registries with 70 DBA patients showed comparable OS between MRD and MUD HCT recipients (91% vs 92%). However, in patients that underwent HCT after year 2000, recipients of MUD HCT had slightly lower chronic graft-versus-host disease (cGVHD)-free survival (GFS) compared to MRD HCT recipients (87% vs. 100%, p=0.06) (47). Similar observations about comparable OS after MRD HCT and MUD HCT were made by the European Society for Blood and Marrow Transplantation (EBMT) and Italian Association of Pediatric Hematology and Oncology Registry (**Table 1**), although GFS was not reported (41, 46). Overall, these data show that 1) MRD HCT outcomes continue to be excellent and 2) OS after MUD HCT is comparable to those after MRD HCT. In the following sections, we will discuss more specific factors affecting outcomes after HCT in DBA.

**Table 1:** Summary of results from key DBA HCT studies since year 2000.

Study, year, and country	MRD, OS	Alternative Donor, OS	Conditioning regimen (number of patients)	Graft failure (number of patients)	aGVHD (II-IV)	cGVHD
Vlachos et al, 2001, USA (37)	N=8; 87.5%	N=12; 14.1%	Bu/Cy – 12 TT/Cy -3	1	ND	ND
Roy et al, 2005, USA (38)	N=41; 76%	N=20; 39%	BU/Cy – 44 Cy/Rad - 13	6	28%	26%
Lipton, 2006, USA (26)	N=21; 72.7%	N=15; 19.1%	ND	1	ND	ND
Mugishima et al, 2007, Japan (39)	N=8; 100%	N=11; 81.8%	Bu/Cy – 8 Cy/Rad - 11	2	25%	6.3%
Vlachos et al, 2010, USA (27)	N=ND 90% (<9 years old) 70% (>9years old)	N=ND 23.1% (prior 2000) 85.7% (since 2000)	ND	ND	ND	ND
Bizzetto et al, 2011, EBMT (40)	N=13 (UCBT); 100%	N=8 (UCBT); 37.5%	ND	0	ND	ND
Fagioli et al, 2014, Italy (41)	N=16; OS 80.4%	N=14; OS 69.9%	Bu/TT/Flu-15 Bu/Cy – 4 Treo/TT/Flu-4	1	41%	21%
Strahm et al, 2018, France and Germany (42)	N=45; 91%	N=25; 92%	Bu/Cy – 35 Treo/TT/Flu – 13	1	24%	11%
Junior et al, 2020, Brazil (43)	N=25; 80%	N=19; 55%	Bu/Cy – 25 Bu/Flu - 15	8	25%	20%
Behfar et al, 2019, Turkey (44)	N=9; 77.8%	N=1; 100%	Bu/Cy – 10	1	60%	10%
Koyamaishi et al, 2021, Japan (45)	N=5; ND	N=22; ND	Bu/Cy – 12 Mel/Flu – 9	0	48.1%	33.3%
Miano et al, 2021, EBMT (46)	N=58; >80%	N=37; >80%	Bu/Cy – 47 Bu/Flu – 16 Treo/Flu – 15	5	30%	15%

N, number of patients; MSD, matched sibling donor; MMD, mismatched donor; OS, overall survival; aGVHD, acute graft versus host disease; cGVHD, chronic graft versus host disease; UCBT, umbilical cord blood transplant; Bu, Busulfan; Cy, Cyclophosphamide; Flu, Fludarabine; Treo, Treosulfan; TT, Thiotepa; Rad, radiation; ND, not documented.

### Pre-transplant factors

One of the major questions facing DBA patients and caregivers is to decide on when to undergo and how to prepare for allogeneic HCT. Several studies suggest better outcomes in younger patients (age < 10 years) compared to older patients (41–43, 46). While some of the newer data show improvements in OS for older patients, the incidence of cGVHD and transplant-related mortality (TRM) rates remain high. This is likely due to a higher transfusion exposure leading to iron-related organ toxicity, potentially development of donor-specific antibodies, and increased interval between diagnosis and transplant – all of which could affect organ function. Additionally, there is some evidence to suggest DBA is a pro-inflammatory condition (9, 48, 49), which could contribute to the end-organ damage and poor TRM in older patients. Therefore, HCT is recommended before age 10 years in the most recent EBMT guidelines (36). Iron overloading also increases the risk of sinusoidal obstruction syndrome (SOS) or hepatic veno-occlusive disease (VOD), especially after busulfan-based myeloablative conditioning (MAC) regimens (45). Therefore, DBA patients require early aggressive and effective chelation which remains a challenge across the world due to lack of experience, restricted access to the drugs, and lack of comprehensive surveillance. For example, it is evident that ferritin is not a reliable biomarker of iron load in DBA patients, which often results in a delay in the initiation of chelation and under-chelation (50–52). Instead, iron content determination by MRI is a widely available and reliable method for iron measurement which utilizes the specific characteristic of iron that shortens T1, T2, and T2* relaxation times. MRI liver and heart are therefore routinely performed and are instrumental in assessing hemosiderosis and guide chelation management in DBA. Imaging-based quantification of other iron-loading organs in DBA such as pancreas or pituitary gland are not available in clinical settings. In some DBA patients, higher non-transferrin bound iron (NTBI), a highly toxic form of reactive iron in blood, is reported even with normal ferritin and liver iron content, suggesting a more complex mechanism of iron overloading and toxicity (50–52). Furthermore, iron overload-mediated damage is often irreversible and not completely corrected by chelation (53). Therefore, iron chelation should be promptly and effectively started earlier in the course of the disease to reduce cumulative iron exposure to reduce organ damage, and not just before an upcoming HCT (52, 53). Deferoxamine, deferasirox and deferiprone are the commonly used iron chelators in DBA. While singe-agent therapy can be used in infants and younger children, combination therapy, such as deferasirox during the day and deferoxamine during night, is often required in older transfusion-dependent patients for effective chelation. Deferiprone is typically reserved for patients with severe cardiac iron loading and/or heart dysfunction. EBMT recommends to reduce liver iron content (LIC) to ≤ 2 mg/g dry weight before HCT (36). However, based on our experience and the international standard among DBA experts, LIC values should be optimally lowered before HSCT to be as close as possible to 3mg Fe/g and not exceed 7mg/g, although due to rarity of the disease and lack of randomized trials, there is no evidence for lowest acceptable LIC values before HCT in DBA. In children with increased LIC levels, our practice has been to perform elastography to assess liver stiffness, which is reliably associated with fibrosis (54).

Other pre-transplant factors to consider are congenital anomalies and organ dysfunction such as cardiac and renal defects, endocrine evaluation, and allo-sensitization and donor-specific antibodies due to multiple prior transfusions (36).

### Conditioning regimen

The current standard is to use MAC regimen for DBA (36). Some expert panels favor treosulfan over busulfan due to reduced toxicities with treosulfan and it remains to be answered in future studies whether treosulfan has clear benefit over busulfan (36, 47). An on-going Blood and Marrow Transplant Clinical Trials Network consortium study in the US is investigating the role of treosulfan based-conditioning for bone marrow failure diseases, including DBA (ClinicalTrials.gov Identifier: NCT04965597). A single-center study from US in patients with BMF disorders transplanted with treosulfan-based preparative regimen (n=23, including 4 patients with DBA) showed a 2-year OS and event-free survival (EFS) of 96%, with a 1-year GVHD-free, EFS of 87% (55).

Reduced-intensity conditioning (RIC) regimens have shown favorable results in several bone marrow failure disorders such as Fanconi anemia and dyskeratosis congenita (56, 57). Limited reports demonstrate good outcomes with reduced-intensity conditioning in DBA, particularly in younger children (< 5 years of age) (45, 58). In a retrospective study of 27 patients with DBA, MAC and RIC regimens had comparable outcomes (OS: 100% vs. 92.9%) (45). Majority of the patients in this study received a melphalan-based RIC therapy (45). This is consistent with laboratory observations where wildtype hematopoietic stem/progenitor cells had a competitive advantage over *RPS19* haploinsufficient cells in mouse and human cell models of DBA, providing rationale for use of RIC regimens (18, 59). Moreover, in the context of poor outcomes in older patients with DBA, use of RIC therapy could potentially be an attractive option for this patient population (27).

Novel non-genotoxic antibody-based conditioning agents have been shown to be effective in promoting engraftment without prominent adverse effects in preclinical models. These involve monoclonal antibodies targeting CD45 or CD117, either naked or conjugated with cytotoxic payloads (60, 61). Preliminary data using CD117-targeting antibody in patients with Fanconi anemia are promising (62). Future prospective studies could explore the role of RIC and antibody-based conditioning in DBA.

### Donor selection

MRDs remain first choice for DBA HCT (27, 36). Importantly, related donors need to be screened for DBA mutations before graft donation. If patient does not have a known DBA mutation, selection of potential related donors relies on clinical and laboratory parameters (i.e., complete blood count, HbF percentage, erythrocyte adenosine deaminase (eADA) level, and potentially bone marrow exam). A previous case report of a persistent erythroid failure in a DBA patient following HCT from a sibling donor with undiagnosed DBA highlights the importance of rigorous screening for DBA in any potential related donors (63).

Outcomes for MUD HCTs have improved tremendously over the last two decades, and OS after MUD HCTs is comparable to MRD HCTs, as described in several studies from Europe (41, 42, 46). However, MUD HCTs are associated with higher cGVHD, especially in children over 10 years of age (42). Therefore, it is key to use shared decision-making after discussion of risk-benefit from MUD HCT for older DBA patients (36).

Unrelated cord blood (UCB) HCTs are associated with inferior outcomes, primarily due to graft failure and higher TRM (39–42, 46). Advances in haploidentical HCT in general have improved accessibility and expanded the pool of donors, but the available evidence for DBA patients remains limited to case-reports (64–66).

### Stem cell source

Bone marrow is preferred over PBSCs whenever possible for non-malignant diseases (36). Additionally, unlike UCB, sibling CB HCTs from sibling donors has shown very good outcomes (42). In one of the larger retrospective DBA studies, 7 sibling CB HCTs were described with 100% OS and one patient with limited cGVHD (42).

### GVHD

Improvements in high-resolution HLA typing has reduced GVHD rates in the recent cohorts. Strahm et al. reported a cumulative incidence of 7% (95% CI, 3-17) for aGVHD (Grade III-IV) and 11% (95% CI, 5-22) for cGVHD (**Table 1**) (42). They also noted that none of the patients who received MRD HCT after year 1999 developed cGVHD. Data from the Italian Registry (n=30, out of which 26 received their HCTs after year 1999), showed slightly higher numbers at 24% for III-IV aGVHD and 21% for cGVHD (n=5, extensive cGVHD in 3 patients) (41).

Retrospective analysis by EBMT showed that iron overloading was associated with extensive cGVHD (24% vs 0%, *P* = 0.04) (46). Other factors that appear to impact cGVHD were occurrence of aGVHD, age of patient (increased risk in older patients), year of transplant (reduced risk in HCTs after year 2000), and donor type (reduced risk with MRD HCTs) (42). Most of the studies have reported calcineurin inhibitor (CNI) with methotrexate (MTX) as the most commonly used GVHD prophylaxis regimen (41, 46). Mycophenolate mofetil (MMF) was used instead of MTX in some cases. A recent report of abatacept added to CNI/MMF combination for unrelated HCTs in bone marrow failure patients looks promising (67). Advances in GVHD prophylaxis have the potential to improve cGVHD-free survival (cGFS) for MUD HCTs in DBA.

### Chimerism

The minimum percentage of donor chimerism to correct erythroid failure in DBA is not known. Also, while partial chimerism could correct anemia, it does not completely eliminate the risk of future MDS/AML. In the Franco-German HCT cohort, 5 patients (10%) were reported to have mixed chimerism without transfusion requirements but the exact chimerism was unavailable (42). In another small study, one patient with 23.5% donor-chimerism was transfusion-dependent, whereas the two others with 76.7% and 54.6% donor-chimerism remained transfusion-free (45). These limited data suggest that a donor chimerism of at least 50% or more is needed to correct erythroid failure after HCT in DBA.

### Long-term monitoring after HCT

While several reports have demonstrated improved outcomes after allogeneic HCT for DBA recently, there is limited data on long-term follow-up of these patients. In addition to the standard long-term follow-up for patients undergoing HCT, DBA patients require additional disease-specific late effects screening (36, 68–70). DBA patients will require follow-up related to prior treatment with corticosteroids, iron overloading and iron chelation agents. This includes vision screening and cataract exams, iron load monitoring using ferritin and/or T2* MRI (preferable) and appropriate management of iron load, evaluation of end-organ damage caused by iron overloading, and hearing screen which can be an adverse effect of certain iron chelators. Patients who are iron-overloaded post-HCT will require routine phlebotomy. DBA patients have several other non-hematological issues, such as craniofacial defects, cardiac, renal, and genitourinary abnormalities which will necessitate multidisciplinary care (27). DBA patients are at slightly increased risk of solid tumors such as osteosarcoma and colorectal cancers, which could be increased by HCT (36). Lastly, DBA patients are at risk for delayed puberty and reduced fertility, which can be further accentuated by exposure to myeloablative chemotherapy used in conventional HCTs (71–73). Therefore, counselling and fertility preservation needs to be offered to all DBA patients considering HCT.

## Gene therapy – progress and challenges

Once *RPS19* was identified as the first DBA gene (followed by discovery of >20 other DBA genes), gene complementation emerged as a potential therapeutic option (74). Although major advances have been made in the last few decades, several preclinical safety, efficacy and regulatory challenges remain before these transformative therapies are ready for clinical use. Autologous HCT of genetically-manipulated HSPCs could overcome several disadvantages of allogeneic HCT, namely a) lack of suitable donors for many patients b) immune toxicities such GVHD c) risk of graft rejection, and d) risk of donor-related hematopoiesis. In general, autologous gene therapy for hematological diseases is carried out by isolating patient HSPCs, genetically correcting them ex vivo, followed by conditioning chemotherapy and infusion of corrected HSPCs (**Figure 1B**). Genetic correction of HSPCs can be achieved using lentiviral vector to express wild type (healthy) copy of the affected gene or using CRISPR/Cas9 or related nucleases for targeted genetic correction of the mutation in the endogenous locus.

Early preclinical work on DBA gene therapies in laboratory models, primarily used retroviral vectors and subsequently used self-inactivating (SIN) lentiviral vectors (75–78). The third-generation SIN lentiviral vectors incorporate several safety features such as deletion of viral enhancer/promoter sequences within the U3 region of 3’ Long Tandem Repeat (LTR), removal of all viral protein genes from the vector plasmid and deletion of viral *Tat* sequence (79). These changes improve safety by reducing the risks of insertional gene activation and replication-competent lentiviral vector generation (79). Preclinical DBA gene therapy studies have primarily focused on *RPS19*, the most common DBA gene (5). One of the major challenges has been limited access to DBA CD34^+^ HSPCs for clinical development, as typically millions of CD34^+^ cells are required for these studies. We recently developed a CRISPR/Cas9-based approach to model *RPS19-*mutated DBA using CD34^+^ HSPCs from healthy donors (18). Utilizing this model, we demonstrated the efficacy of an *RPS19-*expressing SIN lentiviral vector (17, 18). Recently, reduced translation of the erythroid transcription factor GATA1 was suggested to cause the characteristic erythroid defect of DBA, regardless of the mutated RP gene (11). This raises the possibility of forced erythroid-specific expression of *GATA1* as a DBA gene-agnostic therapeutic strategy. Preliminary data using this approach are promising (80).

One of the disadvantages of lentiviral vectors is the inability to control gene dosage in transduced HSPCs. While this is not an issue for RPS19 protein, whose levels are tightly regulated (75), RPL5 and RPL11 overexpression can potentially lead to TP53 stabilization by inhibiting MDM2, a negative regulator of TP53 (81). Therefore, overexpression of RPL5 or RPL11 can potentially lead to TP53-dependent cell death. Hence, targeted approaches such as knock-in of wildtype cDNA cassette following CRISPR/Cas9 editing, precise nucleotide base editing and prime editing need to be explored as future therapeutic options. These novel and promising technologies have shown effectiveness in preclinical setting for other inherited hematopoietic disorders (82–84). Gene correction using knock-in approaches have been shown to be effective in preclinical models of diseases such as chronic granulomatous disease and sickle cell anemia (85, 86). Base editing involves Cas9-nuclease-derived proteins that can precisely convert an adenine to guanine or cytosine to thymine (87). Base editing has been shown to be effective in correcting preclinical models of monogenic diseases such as sickle cell disease (83, 88). However, the diversity of pathogenic mutations that cause DBA also means a single base editor-guide RNA combination can only be used for patients with identical mutations, which poses a unique set of challenges from a preclinical, regulatory, ethical and health economics standpoint. An alternative approach could be prime editing to correct a several adjacent mutations within a short segment of DBA using a single prime editing guide RNA (89). This, for instance, could be a viable approach particularly for *RPS19* between codons 52 and 62, which is a hotspot for several DBA mutations (90). However, it has to be noted that prime editors are less advanced than base editors in terms of preclinical development. Although there are rapid preclinical advances, several practical challenges remain. These include feasibility of mobilization of adequate HSPCs from patients, optimization of lentiviral transduction or genome editing of DBA HSPCs, thoroughly evaluating safety of gene therapy approaches, and regulatory and financial challenges in developing clinical trials for ultra-rare diseases.

In sum, various approaches are being explored towards development of hematological curative therapy for DBA. These approaches could offer a potentially safe and effective therapeutic option for transfusion-dependent DBA patients, especially for those without an MRD (**Figure 1A**). While MRD HCTs have excellent outcome, there is potential clinical equipoise between MUD HCTs and a future gene therapy approach, given the slightly higher rates of cGVHD following MUD HCTs. Furthermore, gene therapy approaches could ultimately prove to be more cost-effective in the long run compared to MUD HCT due to economies of scale. Therefore, the long-term efficacy and safety will have to be determined through carefully designed clinical trials. In particular, it remains to be seen how the risk of MDS/AML is altered following autologous HCT with gene-corrected HSPCs.

## Conclusion and future directions

Overall, outcomes after allogeneic HCT have remarkably improved over the last two decades. This has made HCT a safe and viable option for many patients. However, several unanswered questions and unaddressed challenges remain. Outcomes after HCT in adolescent children is improving but remains suboptimal while there is limited data on HCT outcomes in adult patients with DBA. Even less is known about outcomes in DBA patients who develop MDS/AML or aplastic anemia. Other challenging questions include the roles of reduced-intensity conditioning regimen, antibody-based conditioning approaches and haploidentical HCT in DBA. The risk of solid tumors post-HCT is unknown and long-term follow-up studies are needed. Renewed interest in gene therapy has raised hopes for a safer and effective curative option for select DBA patients. Concurrent advances in our understanding of DBA mechanisms have raised interest and hope in other therapeutic agents such as L-leucine (30) and trifluoperazine (14). Progress in medical therapies including iron chelation have the potential to improve life expectancy and quality of life for DBA patients that are unable to undergo HCT. Therefore, outcomes of patients that undergo HCT and gene therapy will have to be continually evaluated and compared with more current outcome data of non-transplanted patients to determine the risk-benefit of these transformative therapies. The critical need for such continual assessment of natural history and outcomes in a rare disease population highlights the important role played by disease registries, patient advocacy groups and global alliances, and the need for synergy and interaction between the various stakeholders.

## Author contributions

All authors listed have made a substantial, direct, and intellectual contribution to the work, and approved it for publication.
